# Supplementary material for: CRISPR/Cas9-Induced Knockout of Sting Increases Susceptibility of Zebrafish to Bacterial Infection
Source: Biomolecules. 2023 Feb 8;13(2):324. doi: 10.3390/biom13020324 (PMC9953276; doi:10.3390/biom13020324)
Supplement: Supplementary file 1 [file biomolecules-13-00324-s001.zip › biomolecules-2141549-supplementary.pdf]

Supplementary File

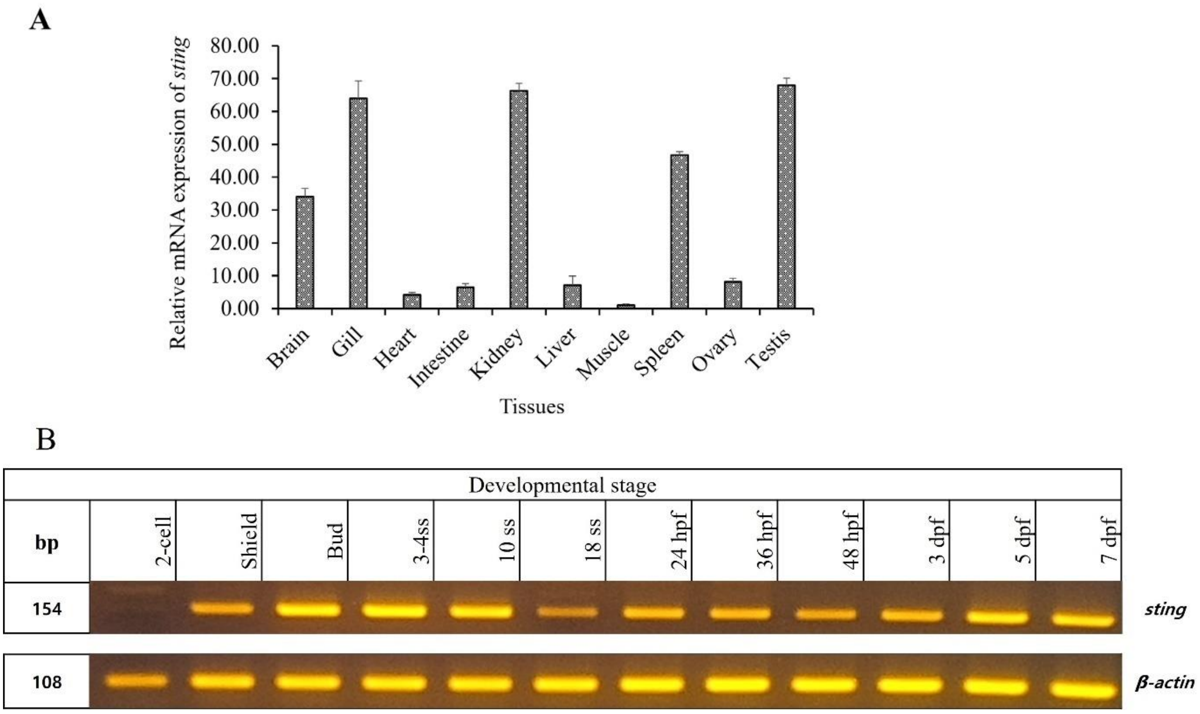

Supplementary Fig. 1. (A) The relative transcription of *sting* in different tissues of healthy adult zebrafish. The data is normalized to the lowest transcription tissue, muscle. The *β-actin* was used as an internal reference gene and the data is shown as the mean of standard deviation (n=3). (B) Transcription of *sting* through the embryonic developmental stages. PCR was performed using the cDNA samples from different developmental stages with *sting* and *β-actin* primers (Table. 1). The PCR product was analyzed by the agarose gel electrophoresis.

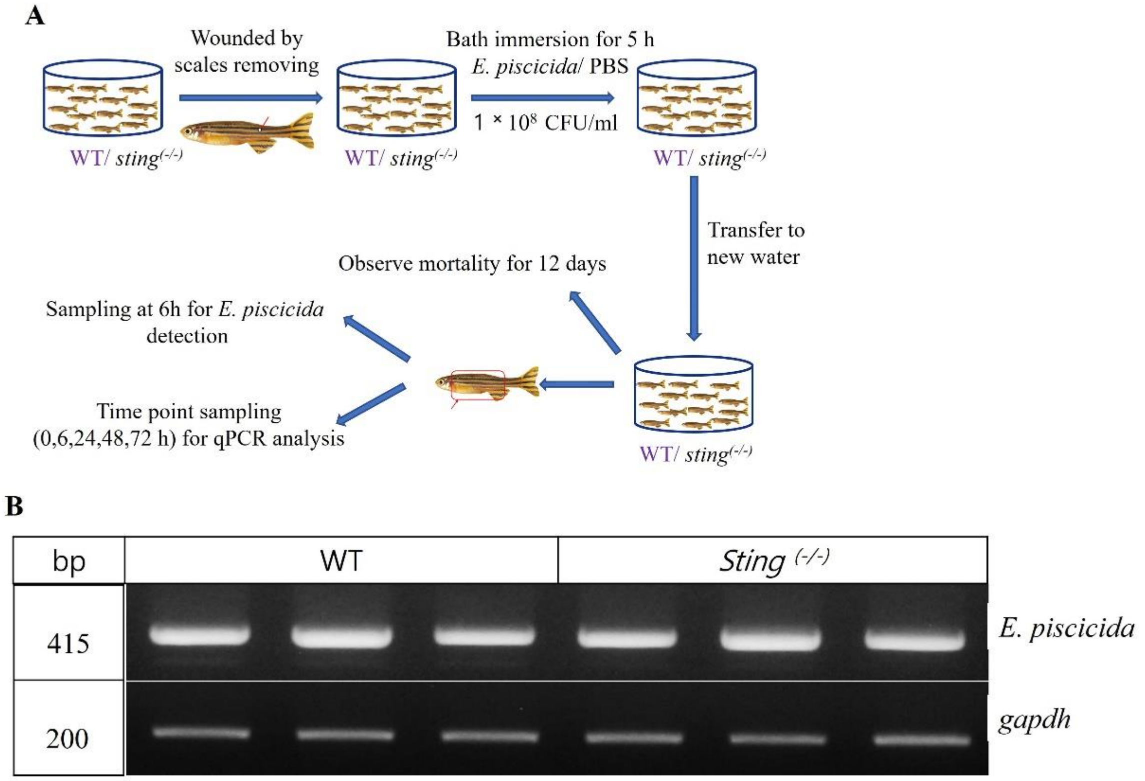

Supplementary Fig. 2. (A) The experimental flowchart of *E. piscicida* infection. The WT and *sting*$^{(-/-)}$ juvenile zebrafish were divided into three groups as mentioned in section 2.5. *The E. piscicida* infection groups and the PBS control groups were wounded by removing 10 scales. The *E. piscicida* at the final concentration of 10^8 CFU/mL was introduced to the *E. piscicida* infection groups and the same volume of PBS was added to the PBS control groups, the fish were transferred to a new tank after 5 hours of infection. Thereafter, the mortality experiment was performed by observing the mortality for 12 days post-infection and the challenge experiment was continued by collecting samples at each time point. (B) PCR confirmation of *E. piscicida* infection at 6hpi. The genomic DNA was extracted from the randomly selected zebrafish samples from WT and *sting*$^{(-/-)}$ and the *E. piscicida* was confirmed by PCR using *E. piscicida* detection primers and *gapdh* primers following agarose gel electrophoresis.
